# Molecular Docking as a Key Driver of Biocontrol for Agri-Food Security

**DOI:** 10.3390/biotech14040080

**Published:** 2025-10-14

**Authors:** María Isabel Iñiguez-Luna, Jorge David Cadena-Zamudio, Marco A. Ramírez-Mosqueda, José Luis Aguirre-Noyola, Daniel Alejandro Cadena-Zamudio, Jorge Cadena-Iñiguez, Alma Armenta-Medina

**Affiliations:** 1 Grupo Interdisciplinario de Investigación en Sechium edule en México, Agustín Melgar 10 Col. Niños Héroes, Texcoco C.P. 56160, Estado de México, Mexico; eunadimiil@gmail.com (M.I.I.-L.); cadenazamudio@gmail.com (D.A.C.-Z.); jocadena@colpos.mx (J.C.-I.); 2 Centro Nacional de Recursos Genéticos-INIFAP, Boulevard de la Biodiversidad 400, Rancho las Cruces, Tepatitlán de Morelos C.P. 47600, Jalisco, Mexico; ramirez.marco@inifap.gob.mx (M.A.R.-M.); aguirre.jose@inifap.gob.mx (J.L.A.-N.); armentam.alma@inifap.gob.mx (A.A.-M.); 3 Colegio de Postgraduados, Campus San Luis Potosí, San Luis Potosí C.P. 78620, San Luis Potosí, Mexico

**Keywords:** molecular docking, agri-food security, crop pathogens, biopesticides, biocontrol, sustainable agriculture

## Abstract

Molecular docking has emerged as a pivotal computational approach in agri-food research, offering a rapid and targeted means to discover bioactive molecules for crop protection and food safety. Its ability to predict and visualize interactions between natural or synthetic compounds and specific biological targets provides valuable opportunities to address urgent agricultural challenges, including climate change and the rise in resistant crop pathogens. By enabling the in silico screening of diverse chemical entities, this technique facilitates the identification of molecules with antimicrobial and antifungal properties, specifically designed to interact with critical enzymatic pathways in plant pathogens. Recent advancements, such as the integration of molecular dynamics simulations and artificial intelligence-enhanced scoring functions, have significantly improved docking accuracy by addressing limitations like protein flexibility and solvent effects. These technological improvements have accelerated the discovery of eco-friendly biopesticides and multifunctional nutraceutical agents. Promising developments include nanoparticle-based delivery systems that enhance the stability and efficacy of bioactive molecules. Despite its potential, molecular docking still faces challenges related to incomplete protein structures, variability in scoring algorithms, and limited experimental validation in agricultural contexts. This work highlights these limitations while outlining current trends and future prospects to guide its effective application in agri-food biotechnology.

## 1. Introduction

Ensuring global agri-food security has become one of the most pressing challenges of the 21st century, driven by the combined effects of climate change, rapid population growth, and the finite availability of arable land. According to the Food and Agriculture Organization of the United Nations (FAO), more than 820 million people currently suffer from chronic hunger, while crop production faces persistent threats from pests and plant pathogens [1,2]. These biotic stresses not only compromise food availability but also threaten agri-food security, causing substantial economic losses and posing risks to public health [3]. Pathogen-induced crop losses account for a significant reduction in global agricultural output [2], and the growing resistance of these organisms to conventional pesticides has intensified the need for alternative pest control strategies [4]. In addition, the widespread use of synthetic agrochemicals has raised serious environmental and health concerns due to their residual toxicity and long-term ecological impacts. In response to these challenges, molecular docking (MD) has emerged as a powerful computational approach for identifying bioactive compounds with high binding affinities to pathogen-specific proteins [5,6,7]. By enabling the rational design and in silico screening of natural or synthetic molecules, this technique supports the targeted inhibition of key enzymatic pathways in plant pathogens, reducing reliance on chemical pesticides and fostering more sustainable agricultural practices [8]. Such precision not only enhances crop resilience and productivity but also strengthens agri-food security by safeguarding yields against biotic stress while aligning with growing demands for safer, toxin-free agricultural products. Furthermore, MD minimizes the trial-and-error characteristic of traditional pesticide development, thereby reducing both costs and development time. By accelerating the discovery of environmentally friendly alternatives to conventional pesticides, molecular docking contributes to an agriculture that harmonizes productivity, environmental stewardship, human health, and long-term agri-food security [9]. For these reasons, this work explores the importance of applying this chemical-computational tool in agriculture to identify and optimize bioactive compounds with antimicrobial properties, targeting key enzymes in crop pathogens, and outlines current trends, limitations, and future perspectives.

## 2. Search Strategy, Literature Sources and Selection Criteria

To contextualize the role of molecular docking (MD) in agri-food sciences, the literature was surveyed across multidisciplinary sources, primarily Dimensions (https://www.dimensions.ai/ accessed on 10 January 2025) and Google Scholar (https://scholar.google.com accessed on 11 January 2025), which index publications, grants, datasets, clinical trials, patents, and policy documents [10,11]. The search covered 1983–2025 using the query: TI = (molecular docking) AND TI = (agri-food security OR genetic resources OR pathogens OR crops). Boolean operators (“AND”, “OR”) were applied, without restricting results to records whose titles contained all keywords simultaneously. Records were screened by title and abstract to retain peer-reviewed articles and guidelines aligned with the scope of this review; items outside the time window, duplicates, and documents lacking scientific rigor were excluded. The resulting corpus was curated to emphasize contributions most relevant to agriculture and food security. As a descriptive aid to the narrative synthesis, keyword-based visual summaries were generated with VOSviewer (v1.6.20) [12,13,14,15,16] using author- and indexer-assigned keywords from the retained records. Synonymous or repeated terms were harmonized, and only terms occurring at least ten times were displayed to improve readability. In these maps, nodes denote frequent terms and links indicate co-mention; node size approximates frequency, inter-node distance reflects the strength of association, and colors group related terms into thematic clusters. These visualizations are provided solely to support the literature overview and do not constitute a separate methodological or bibliometric analysis.

## 3. Trends in Molecular Docking Research for Agri-Food Security

Molecular docking has increasingly established itself as a transformative tool for addressing challenges in agri-food safety, particularly in the development of sustainable solutions to combat crop pathogens and enhance agricultural productivity. Although its origins lie in pharmaceutical research, the application of this technique in agriculture has grown exponentially, reflecting its adaptability and relevance across various scientific domains [17]. To better understand the evolution and current focus of research in this area, the reviewed literature revealed a total of 4406 publications. From these records, recurrent terms appearing at least ten times were considered, resulting in 33,326 instances that highlight the main thematic axes of the field. This evidence illustrates key trends, major research areas, and the exponential growth of molecular docking studies in agri-food sciences in recent years.

**Figure 1:**
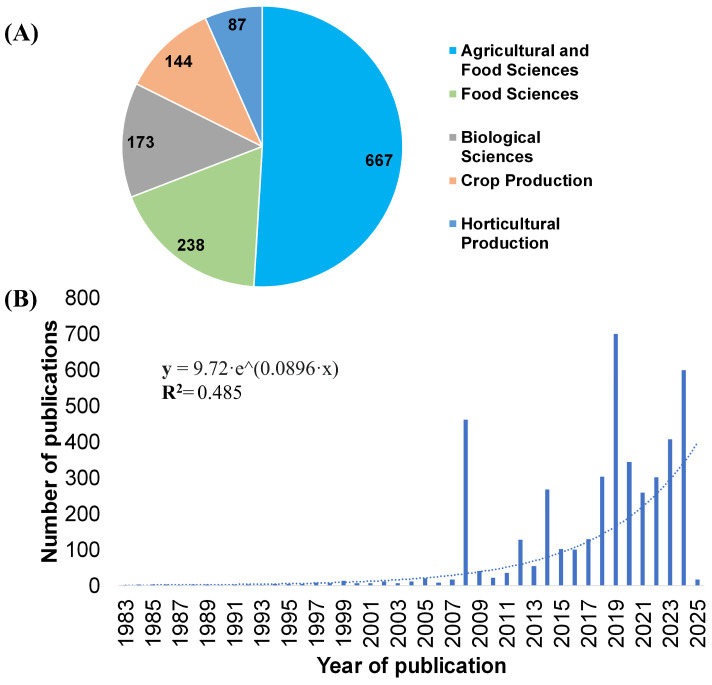
Trends in molecular docking research within the agri-food sector. (**A**) Distribution of publications related to MD across key scientific disciplines. (**B**) Temporal evolution of the number of publications from 1983 to 2025, showing exponential growth in recent years. The trend line is fitted using the exponential model y = 9.72·e^(0.0896·x), with a determination coefficient of R^2^ = 0.485, reflecting sustained growth in the adoption of this technique to address challenges in agriculture and food security.

The distribution of these publications by scientific disciplines showing that most are related to agricultural and food sciences (667 publications), underscoring the centrality of this area in the use of MD (Figure 1A). This is followed by food sciences (238 publications), biological sciences (173 publications), crop production (144 publications), and horticultural production (87 publications). This distribution demonstrates the interdisciplinary nature of the research, with a predominant focus on disciplines directly linked to sustainability and agri-food safety. The temporal evolution of the number of publications from 1983 to 2025 reveals exponential growth in MD research, particularly from 2010 onward, when the number of studies began to increase significantly (Figure 1B). The trend is modeled by the equation y = 9.72·e^(0.0896·x), with a determination coefficient of R^2^ = 0.485, indicating a moderate but consistent correlation. It should be noted that these trends reflect only molecular docking studies aligned with agri-food sciences, and not the global literature across all research domains. This refinement ensures that the analysis remains focused on the agricultural scope of the review. This sustained increase reflects the growing interest in applying this technique to address global challenges such as climate change, pathogen resistance, and the need to sustainably enhance agricultural production. In practical terms, molecular docking contributes to these goals by enabling the rapid identification of plant-derived or synthetic compounds with antifungal or antibacterial activity, guiding the design of stress-resilient cultivars through the discovery of key gene-metabolite interactions, and reducing reliance on broad-spectrum agrochemicals. These applications not only improve crop yield and protection but also promote environmentally friendly strategies aligned with sustainable agriculture.

In addition to these macro-level trends, representative case studies have been compiled, encompassing antifungal and antibacterial bioactives, weed enzyme inhibitors, and nutraceutical leads with functional or preventive properties (Table 1). These examples highlight the breadth of molecular docking applications in agri-food contexts, spanning both crop protection and food sciences.

**Figure 2:**
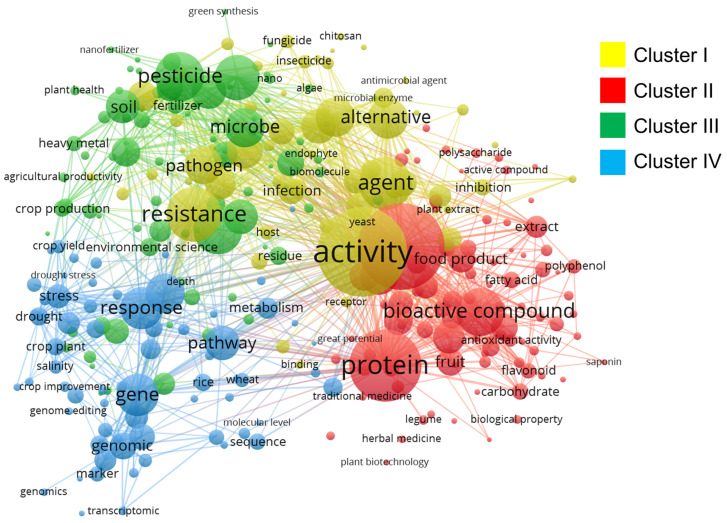
Co-occurrence network map. The thematic grouping of terms related to molecular docking in agri-food research is shown in four clusters. The connections between nodes reflect the interdisciplinary interrelation among these key research areas.

Beyond the compilation of representative case studies, the broader literature research landscape was examined through co-occurrence network analysis, which revealed the thematic structure of molecular docking applications in the agri-food sector. The analysis identified four major clusters (I–IV), each representing a distinct but interconnected research focus that underscores the multidimensional nature of current investigations. Cluster I (Figure 2) is characterized by terms such as activity, agent, alternative, inhibition, and pathogen, emphasizing the development of antimicrobial agents and alternative bioactive compounds. A particular focus is placed on natural molecules to combat crop infections, with strong associations with concepts such as antimicrobial agent, fungicide, and biomolecule. This reflects a shift toward sustainable solutions where synthetic pesticides are replaced by compounds with lower environmental impact, signaling an emerging trend toward integrated chemical and biological strategies for agricultural disease management. Cluster II (Figure 2), dominated by terms including bioactive compound, protein, fruit, extract, and antioxidant activity, is oriented toward the exploration of plant-derived compounds with significant biological properties. This encompasses antioxidants, polyphenols, and flavonoids, which have dual applications in crop improvement and in enhancing human nutrition through functional food production. This cluster illustrates a synergistic connection between agriculture and the food industry, with the potential to deliver agricultural products that combine productivity with added health benefits. Cluster III (Figure 2) comprises terms such as resistance, microbe, pesticide, soil, and pathogen, reflecting research on crop resistance and plant-microorganism interactions. The presence of terms like environmental science and fertilizer suggests a sustainability-oriented approach, exploring how the interplay between plants, soil, and microorganisms can be harnessed to reduce reliance on chemical pesticides and improve soil health. This cluster reinforces the importance of integrating ecological and microbiological perspectives for holistic agricultural problem-solving. Cluster IV (Figure 2) is associated with terms such as gene, response, genomic, pathway, and crop plant, capturing research focused on genomics and molecular biology. It highlights the adoption of advanced tools such as CRISPR-based gene editing and multi-omics approaches to investigate and enhance crop responses to stress factors, including drought, salinity, and other climate change-related challenges.

The interaction among the four clusters underscores the inherently interdisciplinary nature of molecular docking research applied to agriculture. Advances in the identification of bioactive compounds (Cluster II) can be combined with genomic approaches (Cluster IV) to enhance crop resistance (Cluster III), while simultaneously guiding the development of sustainable antimicrobial agents (Cluster I). This interconnected framework reflects a growing integration of chemistry, biotechnology, and agricultural sciences, positioning molecular docking as a pivotal tool to address complex challenges in food production, environmental sustainability, and climate change mitigation. Within this context, the co-occurrence network not only delineates established research domains but also reveals opportunities for innovation. For instance, the development of alternative agents in Cluster I could be accelerated through genomic tools described in Cluster IV. Likewise, research on plant–soil–microorganism interactions (Cluster III) could be enriched by the bioactive compound discoveries highlighted in Cluster II, leading to integrated agricultural management strategies that merge ecological and biochemical approaches. These interconnections demonstrate how molecular docking, when coupled with complementary scientific disciplines, can deliver innovative, sustainable, and impactful solutions to pressing agricultural problems. Overall, the findings highlight both the thematic diversity and the transformative potential of molecular docking in agri-food research, while emphasizing its capacity to address multifaceted challenges from a truly interdisciplinary perspective. Given the diversity of research topics and the critical role of MD in addressing agricultural and food safety challenges, it is necessary to examine the fundamental principles underlying this technique. A clear understanding of its methodological framework helps explain how it can support the trends identified above by enabling the discovery of novel bioactive molecules, guiding genetic improvement strategies, or designing targeted antimicrobial agents. By delving into the key operational steps of MD and the rationale for its application in the agri-food sector, we can better appreciate how it bridges laboratory research and real-world agricultural innovation.

## 4. Fundamentals of the Technique and Its Application in Agri-Food Security

**Figure 3:**
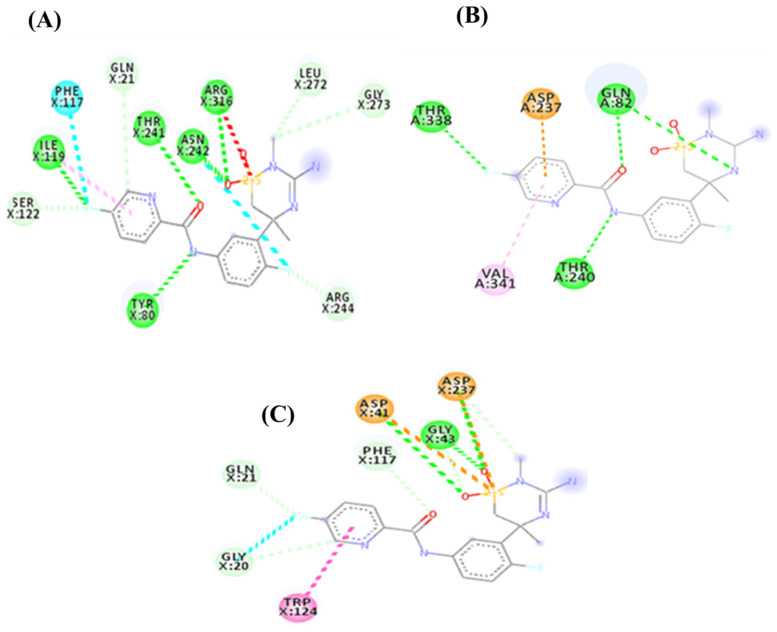
Estimation of interactions in the enzyme–ligand complex through molecular docking. (**A**) Crystallographic structure of the enzyme–ligand complex. (**B**) Enzyme–ligand complex with a different three-dimensional arrangement of the ligand. (**C**) Enzyme–ligand complex with changes in the three-dimensional arrangement of both the ligand and the enzyme.

To contextualize the thematic trends previously described, it is essential to understand the basic principles of molecular docking and its contribution to sustainable solutions in agri-food systems. In essence, molecular docking predicts the binding position and affinity of a ligand to a macromolecule such as a protein by modeling their potential interactions. This virtual screening process enables the evaluation of numerous compound-target combinations, greatly reducing the need for costly and time-consuming laboratory experiments [21,22,23]. The docking workflow typically comprises two fundamental phases. The first is the conformational exploration of the ligand within the macromolecule’s active site, generating a set of possible complex conformations [24,25]. The second involves evaluating these conformations through scoring functions to identify the one with the highest binding affinity, thereby ranking the most promising candidates for further experimental validation [6,23]. Given that conformational searches yield a large number of potential solutions, scoring functions play a critical role in distinguishing biologically relevant conformations from irrelevant ones. They assess multiple properties such as intermolecular interactions, desolvation, and electrostatic and entropic effects [6,23,24] and are generally classified into three categories: force field-based, empirical, and knowledge-based functions, each with particular strengths for optimizing ligand selection (Figure 3) [6].

**Figure 4:**
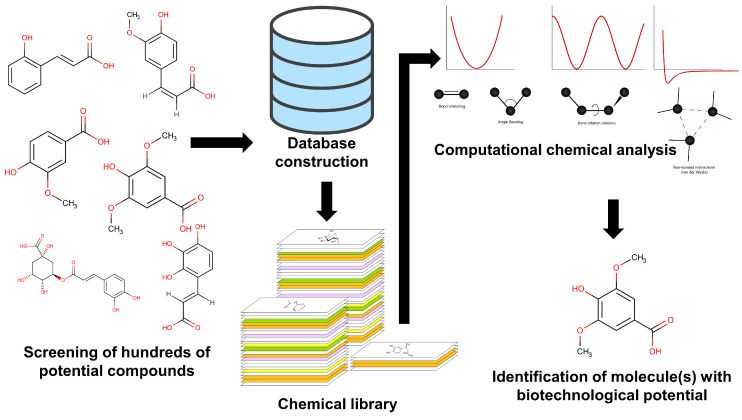
Workflow for the selection, analysis, and identification of natural-origin molecules with biotechnological potential for use in the agri-food sector.

This methodological precision is directly relevant to crop disease control, enabling the design of more effective agricultural practices and strengthening food security [26,27]. One notable advantage is the ability to conduct large-scale virtual screenings that can analyze thousands of compounds within short timeframes (Figure 4) [17]. This capability is particularly valuable in the agri-food sector, where rapid identification of bioactive molecules such as biological pesticides or biocontrol agents can have an immediate impact on reducing chemical pesticide use, improving crop resilience, and safeguarding food supply chains [27]. Consequently, virtual screening through molecular docking emerges as a fundamental tool for agri-food security research, providing critical insights into the mechanisms of action of diverse molecules and informing the development of sustainable, science-driven interventions.

## 5. Molecular Docking of Natural Origin Molecules and Nanoparticles for Sustainable Crop Protection

**Figure 5:**
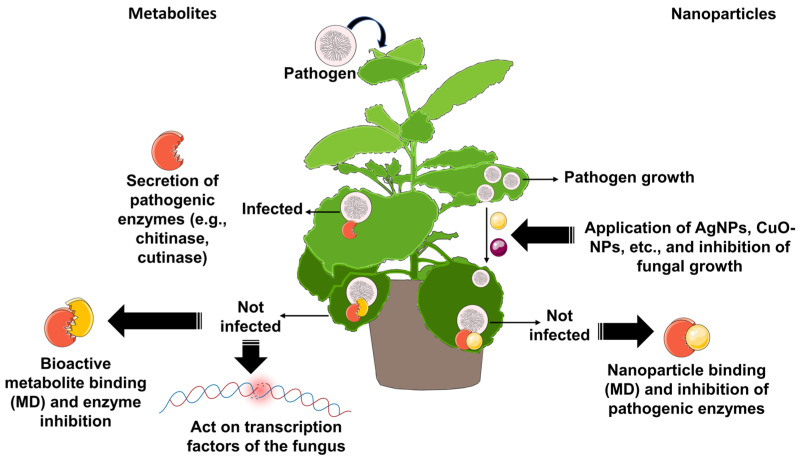
Conceptual representation of pathogen infection and its inhibition by natural metabolites (**left**) or green-synthesized nanoparticles (**right**). Pathogens release key enzymes required for infection, which can be blocked either by bioactive compounds acting on enzymes or transcription factors, or by nanoparticles interacting with pathogenic enzymes, as supported by molecular docking (MD) analyses.

The use of natural molecules in controlling crop pests and diseases offers several advantages for both agri-food security and agricultural sustainability [28]. Unlike chemical pesticides, which often leave harmful residues in food and the environment, natural molecules derived from plants or microorganisms are biodegradable and less damaging to ecosystems [29,30]. One of the main concerns in food security is the contamination of agricultural products with chemical residues [28]. Conventional pesticides, although effective, can persist in food even after processing, posing a potential risk to public health [31]. In contrast, natural compounds identified through molecular docking techniques have more specific actions and degrade more rapidly, significantly reducing residue levels in food [26]. This not only improves the quality and safety of products but also increases their value in the global market, where consumers increasingly demand chemical-free food. Furthermore, the use of biopesticides derived from natural molecules promotes more sustainable agriculture, as they act selectively on pathogens without negatively affecting beneficial soil microorganisms. This is essential for crop health and long-term productivity, maintaining fertile soils and contributing to agricultural sustainability (Figure 5) [32,33]. Several studies exemplify how molecular docking can guide the identification of natural-origin molecules with antifungal and stress-mitigation properties. For instance, in finger millet (*Eleusine coracana* (L.) Gaertn.), docking-based analysis of ABA receptors (PYL proteins) and NF-Y transcription factors revealed critical interactions that regulate drought- and salinity-response pathways [34]. Likewise, in *Fusarium oxysporum*, allyl isothiocyanate from mustard essential oil showed strong in vitro inhibition, consistent with docking-predicted binding to chitin synthase [35]. In tomato wilt caused by *F. oxysporum* f. sp. *lycopersici*, curcuminoids from turmeric were docked to the cutinase active site, correlating with reduced enzymatic activity in vitro [36]. For *Rhizoctonia solani*, docking-guided prioritization of compounds against the calcineurin-responsive transcription factor Crz1 highlighted stress-response pathways as tractable antifungal targets [37]. Similarly, CuO nanoparticles synthesized from *Heliotropium bacciferum* exhibited high growth inhibition of *R. solani* and *F. oxysporum*, with docking results supporting interactions with chitin synthase and cutinase [38].

Another significant advancement has been the development of green-synthesized metallic nanoparticles (e.g., AgNPs, CuO-NPs) from plant extracts (Figure 5). Their antifungal activity has been consistently demonstrated in vitro, for instance with silver nanoparticles tested against *R. solani* [39] and biosynthesized AgNPs from beech bark extract tested against Candida albicans [40]. Moreover, recent reviews emphasize that, beyond in vitro inhibition assays, several studies have also confirmed antifungal efficacy in situ, reducing disease severity when nanoparticles were applied directly to crops under greenhouse or semi-field conditions [41]. Using molecular simulation tools, recent studies have examined how biogenic metal-oxide nanoparticles can interact with chitin synthase (CHS) and other fungal targets; for example, CuO-NPs derived from plant extracts showed favorable docking poses and binding energies within the catalytic vicinity of CHS from *R. solani*, *F. oxysporum*, and *Botrytis cinerea*, supporting a plausible CHS-targeted mode of action [38]. The essential role and druggability of CHS are further supported by cryo-EM structures from *Candida albicans* and *Phytophthora sojae*, which resolve substrate and inhibitor (nikkomycin Z, polyoxin D) binding and provide an atomic-level framework to interpret docking results [42,43]. Collectively, these in vitro and in silico findings indicate strong antifungal potential for green-synthesized nanoparticles and illuminate credible molecular mechanisms underlying their activity [38,39,42].

This study and many others provide compelling evidence of how molecular docking can guide the discovery of bioactive natural molecules and nanoparticles with potential agricultural applications (Table 2). Such integrative approaches not only contribute significantly to crop protection but also open new opportunities for implementing more sustainable control methods. Instead of relying exclusively on traditional chemical pesticides, eco-friendly nanoparticles and natural-origin molecules emerge as alternatives that can be tailored to different pathogen strains and production systems.

These studies significantly advance crop protection while paving the way for more sustainable pest management strategies. By replacing conventional chemical pesticides with eco-friendly nanoparticles and natural-origin molecules, it becomes possible to target pathogens more selectively and minimize environmental impact. Beyond their immediate applications, molecular models also provide the flexibility to design customized strategies for diverse agricultural regions, which has already been demonstrated in key agri-food crops where docking-guided analyses have uncovered promising candidates for disease resistance and stress tolerance.

## 6. Applications of Molecular Docking in Major Agri-Food Crops

Molecular docking has become a valuable tool in agricultural research, applied to major crops such as rice, wheat, sorghum, and maize to unravel mechanisms of resistance to biotic and abiotic stresses. Its integration with molecular dynamics and experimental validation has expanded opportunities for sustainable crop protection and genetic improvement.

### 6.1. Rice

In rice (*Oryza sativa* L.), a staple food for more than half of the global population, molecular docking has clarified how bioactive compounds and defense-related proteins interact to strengthen immunity. For example, immunopeptides obtained from enzymatic hydrolysis were modeled against MHC-II molecules, revealing stable hydrogen bonding with key residues and improved binding after glucose modification [51]. This provides a theoretical basis for designing peptides that enhance immune responses and reduce dependence on pesticides. Likewise, docking studies on WRKY proteins demonstrated their binding to promoter regions of resistance genes (Pi2, Pi54) under drought and blast stress, highlighting the potential of OsWRKY88 and OsWRKY102 as molecular targets for improving blast resistance [52]. The findings revealed differential binding patterns, highlighting that the proteins OsWRKY88 and OsWRKY102 exhibited reduced transcription levels under combined drought and blast stress conditions. These proteins showed significant interactions with the zinc finger residues of the resistance genes, providing a molecular framework for developing targeted genetic strategies to enhance disease resistance in rice varieties [52]. Additional analyses revealed interactions between salicylic acid and the resistance protein OsAAA1, offering insights into biochemical defense mechanisms and guiding strategies for engineering disease-resistant rice cultivars [53]. More recently, in silico docking combined with structural modeling pinpointed a specific mutation in *Magnaporthe oryzae* succinate dehydrogenase MoSdhB^H245D that reduces benzovindiflupyr binding and confers resistance; this mechanism was corroborated by SDH activity assays and site-directed mutagenesis, with positive cross-resistance to other SDHIs and no detectable fitness penalty, informing resistance-management strategies [54]. In parallel, structural and docking-guided studies on blast effectors show that AVR-PikD binds and stabilizes rice sHMA proteins (OsHIPP19/20), promoting effector-triggered susceptibility; these insights dovetail with structure-guided engineering of HMA-integrated NLRs (Pik-1) to broaden recognition specificity against AVR-Pik variants [55,56].

### 6.2. Wheat

In wheat (*Triticum aestivum* L.), MD has been particularly useful for dissecting resistance to fungal pathogens such as *Puccinia graminis*. Genetic diversity analyses combined with in silico predictions have differentiated resistant and susceptible genotypes, supporting breeding programs [57]. Molecular docking also elucidated the interaction between TaPR1a and TaLTP3 proteins, which activate hormonal signaling and strengthen defenses against leaf rust [58,59]. Recent studies confirmed the resistance mechanism to ipconazole in *Fusarium pseudograminearum*, where mutations in CYP51 altered fungicide binding affinity [60]. And recent studies confirmed that resistance to the demethylation inhibitor ipconazole in *Fusarium pseudograminearum* is mediated by the G464S mutation in FpCYP51B, which reduces the binding affinity of the fungicide. Molecular docking validated this conformational change, showing how the mutation weakens hydrophobic interactions in the binding pocket and explains the observed cross-resistance to other triazoles [60]. In parallel, docking analyses of new pyrazole-4-carboxamide and spirooxindole derivatives against *F. graminearum* succinate dehydrogenase identified substitutions at the phenyl and oxindole rings that improved binding energies and inhibitory activity, pointing to these scaffolds as promising fungicide leads. These results complement structural insights into the TaPR1a-TaLTP3 complex, where docking revealed the stabilization of PR1-LTP interactions and their impact on hormonal signaling cascades, reinforcing the molecular framework underlying wheat defense responses [61].

### 6.3. Sorghum

In the case of sorghum (*Sorghum bicolor* L.), MD studies have revealed the molecular basis of stress responses. A single mutation in the SbCYP71A1 protein affects melatonin synthesis. Through modeling the interactions between SbCYP71A1 and its substrate, tryptamine, significant structural alterations were revealed that negatively impacted photosynthetic efficiency, leaf thickness, and vascular development, resulting in reduced biomass [62]. Other studies showed how melatonin binds to antioxidant enzymes (APX, CAT, POX, SOD), enhancing their activity under salt stress and improving ionic balance [34]. For pest control, proteinase inhibitors (CanPIs) from *Capsicum annuum* were modeled against digestive enzymes of the sorghum stem borer (*Chilo partellus*), with CanPI-7 displaying strong inhibitory activity and leading to reduced larval growth and fecundity [63]. More recently, structural and docking analyses of PYL (PYR1-like) receptors in *S. bicolor* clarified their role in ABA perception and drought tolerance. Modeling revealed conserved hydrophobic pockets for ABA binding and specific amino acids responsible for stabilizing receptor–ligand interactions, which were validated through molecular docking and MD simulations. These insights highlight how sorghum PYLs regulate stomatal closure and water-use efficiency under drought stress [64]. Complementary, docking-guided studies with antifungal peptides from solanaceous plants (AMPs) revealed strong interactions with *Rhizoctonia solani* virulence factors such as chitin synthase and glucanase. The computational predictions were corroborated by in vitro inhibition assays, which showed significant reduction in mycelial growth and sporulation. Molecular dynamics (100 ns) further validated the stability of peptide-enzyme complexes, highlighting AMPs as promising biocontrol agents with potential application in sorghum pathosystems [65].

### 6.4. Maize

Lastly, in maize (*Zea mays* L.), docking analyses of Hm1/Hm2 resistance genes identified key residues involved in detoxifying HC-toxin from *Cochliobolus carbonum*, clarifying the molecular basis of resistance [66]. Other docking-guided studies showed that compounds from bergamot oil (linalool, linalyl acetate) interact with antifungal proteins and combined with chitosan emulsions, improve seed protection and germination against *Fusarium oxysporum* and *Xanthomonas campestris* [67]. Using the same approach, ACCA, an ethylene precursor, was identified as a key compound for improving maize resistance to biotic and abiotic stresses, with high binding affinity to the UGT706F8 protein enhanced maize’s defensive responses by modulating ethylene signaling, improving drought tolerance and pathogen resistance [68]. For pest control, molecular docking identified compounds from the essential oil of *Chrysanthemum parthenium* (L.) Sch.Bip., such as 1,6-dioxaspiro [4,4] non-ene and β-farnesene, with high affinity for acetylcholinesterase (Dm AChE), surpassing synthetic insecticides like malathion. These compounds achieved 100% mortality of maize weevil (*Sitophilus zeamais*) at low concentrations, standing out as promising natural fumigants for sustainable and safe postharvest management [69]. Beyond insect control, structure-guided docking against *Aspergillus flavus* identified novel AflG inhibitors that disrupt aflatoxin biosynthesis, providing a rational path to reduce maize grain contamination [70]. For mycotoxin detoxification on infected maize grains, laccase (PDB 1HFU) and manganese peroxidase (PDB 1MNP) showed favorable docking interactions with aflatoxin B1 and deoxynivalenol, consistent with measured in vitro degradation and highlighting an enzymatic bioremediation route [71].

Beyond theoretical predictions, several molecules identified by docking have advanced into agricultural use. Azadirachtin, a limonoid from neem (*Azadirachta indica* A.Juss.), has been modeled against insect receptors and is now a validated biopesticide. Similarly, triazole fungicides such as ipconazole, first studied in silico for CYP51 inhibition, are widely applied in cereals and maize [60]. More recently, metabolites from *Trichoderma* spp., including trichotetronine and bisorbibutenolide, showed strong binding to the rice blast enzyme GSK-1, with simulations and preliminary in planta tests supporting their antifungal potential [20]. These cases highlight how docking can progress from prediction to practice when coupled with experimental validation.

Together, these examples highlight molecular docking as a powerful approach for identifying natural compounds, enzymes, and biocontrol strategies that enhance resistance and improve pest and toxin management in maize. More broadly, docking has proven invaluable for advancing the understanding of the molecular bases of disease resistance and the functional properties of crops of agri-food interest [25]. By revealing interactions at the molecular level, it not only supports crop improvement but also the design of products with potential benefits for human health, thereby connecting theoretical modeling with practical applications. This integrative perspective has driven significant progress in genetic engineering, pest management, and crop enhancement [6]. while at the same time exposing key technical challenges and limitations that must be addressed to fully realize its potential in agricultural biotechnology [72].

## 7. Technical Challenges and Computational Limitations in Molecular Docking

The application of molecular docking in agricultural and food sciences, while promising, faces important technical and computational challenges that must be addressed to ensure reliable and reproducible outcomes [73]. These limitations arise from the intrinsic complexity of biological systems, the variability of environmental conditions, and the demand for high computational precision in order to generate biologically relevant predictions [74]. In addition, the scarcity of high-resolution structural data for plant-specific receptors, resistance proteins, and microbial effectors often restricts the broader adoption of this approach. Addressing these barriers is critical to fully unlock the potential of molecular docking as a transformative tool for agricultural innovation [5].

### 7.1. Ligand Conformation and Flexibility

A central challenge in docking lies in the intrinsic flexibility of ligands. The conformational search strategy dictated by the docking algorithm strongly influences outcomes. Systematic searches provide exhaustive sampling but are computationally expensive, often generating a combinatorial explosion of conformers [25]. Random or stochastic searches, while more efficient, may miss biologically relevant binding modes. This trade-off is particularly critical in agriculture, where large-scale screenings of diverse plant secondary metabolites (e.g., flavonoids, terpenoids, alkaloids) are common. Because these compounds typically exhibit multiple rotatable bonds, docking engines may fail to capture the bioactive conformations that truly interact with pathogen enzymes, leading to underestimation of their antifungal or insecticidal potential.

### 7.2. Protein Flexibility

Proteins also exhibit dynamic behavior, with backbones and side chains adopting multiple conformations. This conformational variability poses significant challenges for accurate protein–ligand and protein–protein docking [75]. Monte Carlo simulations and ensemble docking approaches have shown promise, but wrong-state sampling remains a major source of error [73]. In plant sciences, this limitation is especially relevant for resistance proteins such as NLRs and WRKY transcription factors, as well as fungal enzymes like chitin synthase and cutinase, which often include flexible or disordered domains. Relying on a single static protein structure may therefore oversimplify the complexity of host–pathogen interactions in crops.

### 7.3. Quality of Input Data

Reliable docking depends heavily on the quality of input structures, often derived from Protein Data Bank (PDB) files. However, many PDB entries contain missing residues, unresolved side chains, or inconsistencies that propagate into docking predictions [76]. Preparation modules can partially address these issues, but critical features such as post-translational modifications are frequently overlooked [17]. This limitation is particularly evident in crop sciences, where the scarcity of crystal structures for plant-specific receptors (e.g., ABA receptors, PR proteins) and fungal pathogen enzymes (e.g., necrosis-inducing proteins, effector-binding proteins) forces researchers to rely on homology modeling. Yet, the accuracy of these models depends on the availability of close structural templates, which remain limited in agri-food research.

### 7.4. Scoring Functions and Optimization

Scoring functions are central to evaluating protein–ligand interactions but remain a major source of inaccuracy. Knowledge-based scoring approaches balance speed and interpretability but are limited by assumptions such as the inverse Boltzmann relationship [77]. Hybrid scoring methods that integrate empirical data and machine learning potentials show promise, yet their predictive performance is inconsistent and requires benchmarking against robust datasets [78]. This becomes even more challenging in agri-food studies, where curated datasets for plant proteins or fungal effectors are scarce. As a result, scoring functions originally trained on biomedical targets may not generalize well to crop-specific proteins, such as maize detoxification enzymes or rice blast effectors, reducing the reliability of predictions.

### 7.5. Computational Resources

High-resolution docking with flexible ligands and proteins is computationally demanding. While distributed computing and GPU acceleration have increased feasibility, many agricultural research groups lack access to such infrastructure [76]. Moreover, scaling docking workflows to include natural product libraries from crops and medicinal plants or to screen entire proteomes of fungal pathogens remains a major bottleneck [22]. This is particularly evident in regions where agricultural innovation is most urgently needed, but computational facilities are limited, creating a gap between theoretical advances and their practical adoption in crop protection research.

### 7.6. Statistical Assessment Limitations

Statistical methods such as rank-based consensus scoring or intersection approaches are widely used to refine docking predictions. However, their reliability depends strongly on the robustness of the datasets employed [17]. In the context of agriculture, this problem is exacerbated by the lack of validated reference datasets for plant receptors and pathogen enzymes. For example, docking results of antifungal metabolites against *Fusarium oxysporum* chitin synthase may be misleading if ranked solely by scoring functions without comparison to experimentally confirmed ligands. Without robust benchmark datasets, statistical refinements risk generating false positives that can misguide subsequent experimental validation.

## 8. Biological Challenges and Limitations

Molecular docking, while a powerful tool for drug design and agricultural research, faces biological challenges and limitations that affect its accuracy and applicability. Reliable docking depends heavily on the ability to represent molecular flexibility, yet proteins undergo complex conformational movements that are not always captured in the models [79]. These limitations, while widely acknowledged in biomedical contexts, acquire unique relevance in crop sciences because stress-related proteins often display intrinsically disordered regions or conformational shifts triggered by environmental cues. Additionally, docking relies on high-quality three-dimensional structures obtained through X-ray crystallography, NMR, or modeling, meaning that incomplete or low-resolution structures can yield unreliable results [17]. Preparation modules can partially address these issues, but critical features such as post-translational modifications in plant resistance proteins are frequently overlooked. Docking also presents limitations in representing non-covalent interactions such as hydrogen bonds, hydrophobic interactions, and electrostatic forces, which can lead to inaccurate binding affinity predictions [80]. Another critical challenge is the inability to fully simulate the actual cellular environment, including the presence of other molecules, water, ions, and pH gradients, which affects the validity of results under real biological conditions [76]. This issue is especially evident in plants, where subcellular compartments such as vacuoles, plastids, and the apoplast modulate metabolite concentrations and protein activity. For complex interactions, such as protein–protein interactions or those involving multiple components, molecular docking can be insufficient due to its limited ability to explore all degrees of freedom and find the optimal conformation. Furthermore, current free-energy models lack precision when correlating predicted binding affinities with experimental values, and ligands with novel chemical features often require additional calculations to be parameterized in the software [22]. Finally, molecular docking predictions require experimental validation, which can be both costly and time-consuming. In agriculture, this challenge is exacerbated by the need for large-scale validations under both greenhouse and field conditions, where environmental variability further complicates reproducibility.

### 8.1. Genetic Diversity in Crops

Another aspect to consider is the genetic diversity in crops, which adds significant complexity to molecular docking studies applied to the agri-food sector by introducing variations in the sequences of proteins and secondary metabolites that act as molecular targets [81]. These variations affect the affinity and specificity of the evaluated compounds, making it difficult to generalize results across genetically different varieties of the same crop. This has been observed in species such as common bean (*Phaseolus vulgaris* L.) and wheat, where differences in proteins related to stress or disease resistance complicate the extrapolation of treatments [82,83]. For instance, allelic variants in resistance proteins (e.g., PvPR1 in beans or TaNAC transcription factors in wheat) modify docking outcomes and highlight the limitations of universal models. Furthermore, diversity expands the range of metabolites present in plants, increasing the need for extensive and specific molecular libraries to accurately model molecular interactions, which raises costs and experimental effort [84]. This variability also limits the universal transferability of results, as what works in one variety may not be effective in another, as seen in studies on drought and salinity tolerance in rice and wheat [85]. However, this complexity offers opportunities for more personalized approaches in genetic improvement, enabling the identification of key genetic variants and metabolites that can be used to develop precise and sustainable solutions to address climate change and agri-food demands [86]. In this context, molecular dynamics (MD) studies benefit from specific genomic and proteomic databases but require thorough validations to ensure their applicability under real-world conditions.

### 8.2. Environmental Context

In relation to the environmental context in which plants are cultivated, external factors can significantly restrict the applicability of molecular docking findings, as soil composition, nutrient availability, and climatic conditions deeply affect plant physiology and stress responses. These environmental interactions modulate gene expression, metabolic pathways, and the activity of key proteins, creating discrepancies between results obtained under controlled laboratory conditions and their performance in the field [85]. For instance, in crops like rice and maize, water stress levels and thermal variability can drastically alter the functionality of genes and metabolites previously identified as relevant in docking studies [87]. Moreover, soil microbial composition and plant-microorganism interactions also influence the effectiveness of designed compounds, adding an additional layer of complexity for validation under real-world conditions [88]. This is particularly critical in semi-arid regions, where fluctuating soil salinity and microbial diversity directly alter the availability and stability of ligands and targets predicted in silico.

These limitations underscore the need to develop integrated methodologies that combine docking analyses with experiments in semi-controlled environments that mimic field conditions, to improve the translation of results into practical applications. This requires not only adjustments in experimental design but also innovations in bioinformatic modeling, incorporating environmental variables as part of molecular simulations [89]. The adoption of such holistic approaches is essential to maximize the impact of molecular docking in agriculture, particularly within the context of sustainable and resilient agricultural systems in the face of climate change [90]. In summary, while molecular docking provides an innovative approach to exploring the molecular bases of stress responses in crops, it faces methodological, computational, and contextual challenges that must be addressed to maximize its applicability. Overcoming these limitations will require not only technological advances in algorithms and computational resources but also the integration of more comprehensive omics data and greater consideration of the specific environmental conditions of crops. Ultimately, bridging the gap between in silico predictions and agricultural practices will depend on cross-disciplinary collaboration between computational scientists, plant biologists, and agronomists.

Beyond environmental variability, additional challenges remain intrinsic to molecular docking itself. Technically, the reliability of docking outcomes depends on the quality and resolution of protein structures, the accuracy of scoring functions, and the treatment of ligand flexibility, solvation, and conformational dynamics. Recently, deep learning–based algorithms such as AlphaFold have revolutionized structural biology by predicting three-dimensional protein conformations directly from amino acid sequences. AlphaFold employs attention-based neural networks trained on experimentally resolved structures and large multiple sequence alignments to infer inter-residue distances and angles, providing a per-residue confidence metric (pLDDT) that reflects local reliability. Although the accuracy of AlphaFold is often near experimental resolution for globular and well-folded domains, regions with low pLDDT values usually correspond to intrinsically disordered or conformationally heterogeneous segments. Consequently, these predictions should be treated cautiously when applied to docking, as the single static conformation generated by AlphaFold does not capture conformational ensembles, allosteric transitions, or the dynamic energy landscape of proteins, which may be critical for ligand binding [91,92]. The increasing use of predicted structures, such as those generated by AlphaFold, while valuable, still requires careful curation and validation to avoid artifacts. From a biological standpoint, the complexity of plant–pathogen interactions, the presence of isoforms or allelic variants, and the modulation of signaling pathways under different stresses introduce variability that static docking models alone cannot capture [93,94]. Furthermore, the absence of standardized protocols and benchmarking strategies continues to limit reproducibility across studies. Collectively, these methodological, computational, and biological challenges underscore the need to integrate docking with complementary approaches such as molecular dynamics simulations, omics data, and experimental assays to strengthen its translational applicability in agriculture.

## 9. Future Directions and Perspectives

To address the challenges faced by molecular docking, future research efforts should focus on integrating this technique with other biotechnological approaches, such as genomic editing and transcriptomics, to generate a more holistic understanding of plant stress responses. In this context, the development of standardized protocols that account for variability among different species and environmental conditions could significantly enhance the predictive power and applicability of docking in agricultural science [95,96]. With these advancements, the prospects of molecular docking in agri-food crops appear promising, particularly as computational techniques and molecular dynamics simulations continue to evolve. These technologies not only strengthen the capacity to predict molecular interactions but also accelerate the identification of lead compounds with agricultural applications [18,34]. In a global scenario where food production demands continue to rise due to population growth, molecular docking emerges as a key strategy for designing crops that are more resilient to environmental stresses and have optimized nutritional profiles [53,79].

Beyond stress resilience, molecular docking is also proving valuable in enhancing the organoleptic characteristics of foods, an aspect increasingly relevant to consumer acceptance and market competitiveness. For instance, the metabolomic analysis of Moringa oleifera Lam. leaves using advanced platforms such as UPLC-Q-TOF-MS and GC-MS revealed that compounds including glucosinolates and flavonoids contribute to sweet, bitter, and spicy taste profiles. By elucidating the interactions of these bioactive compounds with taste receptors, docking expands the possibility of optimizing flavor and guiding the development of cultivars with targeted sensory attributes [95]. Similarly, peptides derived from the fermentation of *Stropharia rugosoannulata* mycelium were assessed for their salty and umami properties through molecular docking with T1R1/T1R3 taste receptors. Results showed that peptides with specific sequences interact stably with key residues such as Asp147 and Glu301, generating a pleasant flavor. These insights are particularly relevant for advancing sustainable protein alternatives in the food industry, facilitating the development of plant-based products with improved taste profiles [96]. Together, these examples highlight the synergy between molecular docking and omics technologies in enhancing the sensory perception of foods, ultimately improving consumer acceptance and expanding opportunities for agri-food innovation.

In parallel, molecular docking has also emerged as a complementary tool for enhancing the precision and efficiency of CRISPR-based applications in crop science. By optimizing the interactions between Cas proteins and RNA, docking contributes to improving gene-editing specificity and minimizing off-target effects. Recent computational simulations have modeled conformational states of Cas proteins, including Cas9, providing valuable insights for refining gene-editing systems [97]. Likewise, in silico optimization of RNA-protein interactions in CRISPR systems such as Cas13 demonstrates the potential to design tailored solutions for agricultural challenges, including pest resistance and abiotic stress [98]. A notable example is the CRISPR-assisted editing of the trehalase gene in *A. thaliana*, where docking analyses helped modify substrate-binding domains, conferring drought tolerance. This approach not only enhanced the accumulation of metabolites like trehalose but also improved plant adaptability to adverse conditions, opening avenues for the development of crops resistant to water stress and other abiotic constraints [99].

Recent advances in artificial intelligence (AI) are reshaping docking workflows, transitioning from exhaustive brute-force screening to machine-learning (ML)-augmented pipelines that prioritize the most promising ligands. ML-accelerated docking reduces computational costs by learning from a small subset of docked molecules and then applying predictive models to focus on high-scoring candidates [100]. At larger scales, ultra-large virtual screening frameworks now integrate ML filters and GPU/cloud resources, enabling the adaptive exploration of chemical libraries with billions of molecules while keeping docking tractable [101]. On the modeling front, deep-learning-based predictors of ligand pose and binding affinity, such as graph-transformer architectures, have improved both redocking accuracy and cross-target generalization, complementing classical scoring functions [102]. Moreover, active-learning strategies achieve high recovery rates of top-scoring compounds while docking only a minor fraction of large libraries, making this approach especially attractive when screening structurally complex natural product collections [103].

Molecular docking can also underpin circular-bioeconomy strategies by prioritizing bioactives from agri-food by-products such as peels, pomace, or husks for antimicrobial, preservative, or nutraceutical use, thereby adding value to waste streams while reducing the environmental footprint. Recent reviews emphasize how food-waste valorization can feed sustainable product pipelines and reinforce zero-waste principles within the agri-food sector [104]. As a concrete example, essential oils from citrus peles a classic agro-industrial by-product exhibit strong anti-biofilm and antibacterial activity against Listeria monocytogenes. Their chemically diverse terpenoid profiles are highly amenable to docking-guided target selection [105]. More broadly, valorization frameworks outline routes to convert agro-industrial residues into functional ingredients and biomaterials, positioning docking as a rapid prescreening tool before laboratory extraction and scale-up [106]. Beyond plant-derived streams, peptides from animal processing by-products are already being prioritized through docking prior to purification, providing a transferable model for the efficient valorization of plant-based and mixed agri-food residues.

Crucially, translating in silico insights into real-world agricultural impact requires rigorous experimental validation. Comparative studies have shown that relying exclusively on predicted structures (e.g., AlphaFold models) without curation can compromise virtual screening outcomes compared to experimentally resolved structures, underscoring the need for controls such as redocking co-crystallized ligands and including orthogonal assays Community guidelines stress transparency in reporting, convergence checks, and prospective benchmarking, particularly when progressing from docking to molecular dynamics (MD) and ultimately to wet-lab testing [107]. Such measures are essential to ensure reproducibility, reliability, and translational value in docking-driven agricultural research [105].

Another critical dimension is the inherent gap between computational predictions and real-world biological outcomes. Although artificial intelligence and docking-based pipelines are reshaping discovery workflows, it is well recognized that not all in silico-predicted molecules demonstrate significant activity in experimental assays or clinical trials. From our perspective, these tools should be viewed primarily as hypothesis-generating and prioritization strategies, rather than definitive predictors of efficacy. In the agri-food and biocontrol context, their strength lies in filtering vast libraries to identify the most promising candidates. However, bridging this gap requires multi-tiered validation ranging from molecular dynamics simulations to in vitro and in planta assays, and ultimately field trials supported by integrative omics data. Such an approach not only reduces the incidence of false positives but also enhances the translational value of computational outputs for sustainable crop protection and food innovation.

In line with this reasoning, we emphasize that predictive tools can only yield translational success when combined with robust experimental studies. Computational pipelines therefore represent the first step of a broader validation process, where docking and AI predictions must be systematically complemented with molecular, biochemical, and agronomic assays to confirm efficacy and applicability under real-world conditions.

Ultimately, the integration of molecular docking with MD simulations, CRISPR technologies, and multi-omics approaches represents a transformative step for agricultural biotechnology. This convergence not only accelerates the discovery of new genetic targets but also supports the rational design of crops with enhanced nutritional and sensory qualities, while reinforcing resilience to climate change. In an era of rapidly growing food demands, molecular docking stands as a powerful tool to drive sustainable agri-food systems capable of ensuring productivity, quality, and resilience, while expanding the scientific and technological frontiers of agriculture.

## 10. Conclusions

Molecular docking emerges as a pivotal tool in food safety and agricultural innovation, enabling the discovery of bioactive compounds that enhance pest and disease control while reducing reliance on chemical pesticides. By promoting the rational use of natural molecules, docking contributes not only to minimizing environmental contamination and food toxic residues but also to advancing more sustainable and circular agricultural systems. When integrated with complementary approaches such as CRISPR-based gene editing, transcriptomics, metabolomics, and artificial intelligence-driven predictive models, molecular docking transcends its role as a virtual screening technique to become a cornerstone of next-generation crop improvement. This convergence accelerates the development of resilient cultivars with optimized nutritional and organoleptic qualities, better adapted to the challenges posed by climate change. Crucially, its predictive power must be coupled with rigorous experimental validation to ensure translational value and real-world applicability. Taken together, molecular docking represents a transformative framework for designing crops and food products that are not only productive and safe but also aligned with the urgent need for sustainability and global food security.

## Figures and Tables

**Table 1:** Molecular docking analysis of bioactive compounds identified in different plant species.

Species	Metabolite	Tool	Findings	Validation	Reference
*Puccinia triticina* (wheat leaf rust)	Cynaroside, Prodelphinidin	AutoDock Vina and GROMACS	Cynaroside and Prodelphinidin showed high binding affinity with MAPK1, suggesting their potential as natural fungicides	Molecular Dynamics Simulation (20 ns); RMSD & PCA confirm complex stability	[18,19]
*Chenopodium album*, *Anagallis arvensis*, *Lolium temulentum*, *Echinochloa crus-galli*	Chloroacetamide derivatives	Molecular Operating Environment (MOE)	Chloroacetamide derivatives showed high affinity for VLCFAS, inhibiting fatty acid synthesis in weeds	In planta: EC_50_ by foliar bioassay (SPAD); Docking + Pharmacophore modeling	[19]
*Oryza sativa* (rice)	Trichotetronine, Bisorbibutenolide	Molecular Docking and MD	Metabolites from Trichoderma species inhibited the fungal enzyme GSK-1 of Pyricularia oryzae	Molecular Dynamics Simulations (100 ns)	[20]

**Table 2:** Molecular docking studies identifying bioactive compounds for agricultural pest and pathogen control.

Source	Compound	Pathogen	Tool	Finding	Validation	Reference
*Lecanicillium lecanii*	Bassianolide	*Plutella xylostella*	Molecular docking	Bassianolide showed high toxicity against *P. xylostella* larvae with a mortality rate > 80%. A potent natural insecticide.	In vitro bioassay (3 doses); full chemical characterization (LC-MS, NMR, FTIR); docking study	[44]
*Piper longum* L., *Ocimum gratissimum* L., *Phaseolus vulgaris* L., *Curcuma longa* L., *Inula helenium* Asso	Bisdemethoxycurcumin, rosmarinic acid, chlorogenic acid, piperanine, dihydropiperlonguminine, piperdardine, dihydrocurcumin, longumosides B	*Xanthomonas oryzae* pv. oryzae	AutoDock Vina, GROMACS	Compounds with high binding affinity to peptide deformylase, inhibiting the growth of *X. oryzae*, the cause of bacterial leaf blight in rice.	MD simulation (80 ns), RMSD, MM-PBSA, H-bonds, PCA, ADMET	[26]
*Fragaria vesca* L., *Solanum tuberosum* L., *Vitis vinifera* L.	Phenolic acids (caffeic, chlorogenic, ferulic acids)	*Botrytis cinerea*	AutoDock Vina	Phenolic acids showed high binding affinity with laccase enzyme of *B. cinerea*, suggesting their use as natural inhibitors.	MD simulation	[31]
*Pimpinella anisum* L.	(E)-anethole, Limonene, α-Himachalene, Linalool, trans-Verbenol	*Tribolium castaneum*	Molecular Operating Environment (MOE)	The main compounds of the essential oil and its nanoemulsion showed high binding affinity with ALT and AST enzymes, suggesting a potent insecticidal effect.	GC-MS, in vivo toxicity assay (LC_50_, feeding indices), in vitro enzyme assays (AST, ALT), docking with modeled proteins	[45]
*Syzygium aromaticum* (L.) Merr. and L.M.Perry	Eugenol and its analogs	*Spodoptera frugiperda*	AutoDock Vina, GROMACS, MM-PBSA	Three new eugenol analogs showed high insecticidal activity against *S. frugiperda* larvae (LC_50_ of 0.042 mg/mL by diet and 0.027 mg/mL by topical application).	Homology modeling, MD (50 ns), MM-PBSA, Probit LC_50_ (in vivo), per-residue binding analysis	[46]
*Artemisia vulgaris* L.	Scopoletin	*Amaranthus retroflexus*	Virtual Screening and Molecular docking analysis	Scopoletin showed selective inhibitory activity against weed growth such as *A. retroflexus*.	*In vivo*: plant growth suppression assay (extract-level only)	[32]
*Ocimum basilicum* L.	Chicoric acid, ursolic acid, salvigenin, nepetoidin B, rosmarinic acid	*Rhynchophorus ferrugineus*	AutoDock, ADMET, UPLC-MS/MS	Secondary metabolites inhibited proteolytic enzymes of the red palm weevil. Chicoric acid showed the most insecticidal activity with an LC_50_ of 1132 µg/mL.	Docking + ADMET + in vitro enzymatic inhibition + in vivo larval mortality	[47]
*Solidago graminifolia* (L.) Nutt.	Quercetin, chlorogenic Acid	*Spodoptera frugiperda*	AutoDock Vina, SwissModel, UPLC-MS/MS	Quercetin showed an LC_50_ of 0.157 mg/mL with interactions at the AChE active site. Chlorogenic acid showed no insecticidal activity but antagonized the effect of quercetin.	Docking + homology modeling + UPLC-MS + larval mortality assay (LC_50_) + interaction profiling	[48]
Compounds from ZINC Database	ZINC08952607, ZINC04264850	*Bemisia tabaci*	Virtual Screening, Molecular Dynamics, MM-PBSA	Both compounds showed high binding affinity with the ecdysteroid receptor of *S. frugiperda*, suggesting their use as potential insecticides.	Docking + MD (50 ns) + MM-PBSA + physico-chemical profiling; no in vivo validation	[49]
*Rosmarinus officinalis* Spenn.	1,8-cineole, α-pinene, camphor	*Botrytis cinerea*, *Fusarium oxysporum*, *Alternaria alternata*, *Callosobruchus maculatus*	GC-MS, Glide (Schrödinger v11.5)	Borneol showed the highest activity against insect acetylcholinesterase, and α-caryophyllene against the β-tubulin of *B. cinerea*, with Glide Scores of −7.254 and −7.025 kcal/mol, respectively.	Docking + in vitro antifungal (disk diffusion) + insect bioassay (mortality, oviposition, repellency)	[50]

## Data Availability

No new data were created or analyzed in this study.

## Abbreviations

The following abbreviations are used in this manuscript:

ABA	Abscisic Acid
AI	Artificial Intelligence
CRISPR	Clustered Regularly Interspaced Short Palindromic Repeats
GC-MS	Gas Chromatography-Mass Spectrometry
MD	Molecular Dynamics
ML	Machine Learning
PDB	Protein Data Bank
PR proteins	Pathogenesis-Related Proteins
qPCR	Quantitative Polymerase Chain Reaction
RNA	Ribonucleic Acid
SSR	Simple Sequence Repeat (cuando lo uses en contexto filogenético)
T1R1/T1R3	Taste receptor type 1, subunit 1/Taste receptor type 1, subunit 3
UPLC-Q-TOF-MS	Ultra-Performance Liquid Chromatography coupled to Quadrupole Time-of-Flight Mass Spectrometry
